# Outcome of Endoscopic Transsphenoidal Surgery for Recurrent or Residual Pituitary Adenomas and Comparison to Non-Recurrent or Residual Cohort by Propensity Score Analysis

**DOI:** 10.3389/fendo.2022.837025

**Published:** 2022-04-25

**Authors:** Xuan Gong, Yang Zhuo, Huichun Yuan, Kui Yang, Chuntao Li, Songshan Feng, Mingyu Zhang, Zhenyan Li, Hongshu Zhou, Zhixiong Liu

**Affiliations:** ^1^ Department of Neurosurgery, Xiangya Hospital, Central South University, Changsha, China; ^2^ Department of Neurosurgery, The First People’s Hospital of Changde, Changde, China

**Keywords:** pituitary adenoma, endonasal endoscopic surgery, recurrence, gross total resection, repeat transsphenoidal surgery

## Abstract

**Objective:**

To evaluate the long-term outcomes and safety of endoscopic transsphenoidal surgery (ETS) in recurrent and residual pituitary adenomas (rrPAs), as well as the predictors of gross total resection (GTR) and intraoperative CSF leakage. Furthermore, to compare outcomes and complications with non-rrPAs cohort.

**Methods:**

Clinical and radiological characteristics of patients with rrPAs who underwent ETS were collected between 2017 and 2020. Data of patients with non-rrPAs were collected from 2019 to 2020. Logistic regression analyses were performed to investigate the factors influencing gross total resection (GTR) and intraoperative CSF leakage. Between-group comparisons of outcomes and complications were performed through propensity score analysis.

**Results:**

We enrolled 73 patients with rrPAs. GTR was achieved in 41 (56.1%) cases; further, GTR or near-total resection was achieved in 93.2% of patients. The mean tumor volumes for GTR and non-GTR cases were 6.2 ±7.2 cm^3^ and 11.1 ±9.1 cm^3^, respectively. Multivariate regression analysis of the GTR rate in patients with rrPAs revealed that Knosp grade was an independent factor (odds ratio [OR] = 0.324; p=0.005). Moreover, previous transcranial surgery and non-functional pituitary adenomas were risk factors for intraoperative CSF leakage in patients with rrPAs (OR=6.450, p=0.019 and OR=7.472, p=0.012, respectively). After propensity score matching, There was no significant difference in the GTR rate between patients with rrPAs and patients with non-rrPAs. Contrastingly, patients with rrPAs had a higher rate of intraoperative CSF leakage and longer postoperative hospital stay than patients with non-rrPAs. During the follow-up, vision improved in 9 (22.0%) and 24 (62.5%) patients with rrPAs and non-rrPAs, respectively. Although there was a trend that reoperation of rrPAs involved a lower hypopituitarism recovery rate and biochemical remission rate, as well as a higher hypopituitarism rate, there was no statistically significant between-group difference.

**Conclusions:**

Knosp grade was an independent factor for GTR in endoscopic transsphenoidal surgery in patients with rrPAs. Previous transcranial surgery and non-functional PAs were risk factors for intraoperative CSF leakage. Although associated with longer hospital stay, rrPAs did not associate with lower GTR rate or more frequent postoperative complications than non-rrPAs cohort.

## Introduction

Transsphenoidal surgery is the initial treatment of choice for most non-prolactin-secreting pituitary adenomas (PAs) (1, 2). Since the introduction of endoscopy into transsphenoidal surgery, it has gained acceptance due to allowing better visualization of the supra- and parasellar regions, as well as minimal structures. There have been promising results regarding the tumor resection rates and postoperative complications after endoscopic transsphenoidal surgery (ETS) (3–7). However, PAs are likely to recur even after complete resection, especially in the long term. The reported overall recurrence rate after gross total resection (GTR) is 7%–33%, with cases of incomplete resection showing higher recurrence rates (8–12). Risk factors for recurrence include labeling index, cavernous sinus invasiveness, and young age at diagnosis (9, 13, 14).

Radiation therapy and medication are relatively safe treatment options for recurrent PAs (15, 16). However, repeat surgery is often preferred when the tumor is very large, close to optic chiasm or hormone secreting (17–19). Repeat surgery for recurrent or residual PAs (rrPAs) is technically challenging due to the absence of anatomical landmarks and presence of scar tissue. Compared with initial surgeries, re-operative pituitary surgeries have more frequent complications and a lower GTR rate (20–23). However, few studies explored factors affecting GTR or risk factors for complications of re-operative pituitary surgery. Perhaps even less studies compared the outcomes of ETS for rrPAs with those for non-recurrent or residual pituitary adenomas (non-rrPAs). This study aimed to describe a series of cases of rrPAs resected through ETS and to explore factors related to GTR and intraoperative CSF leakage. Furthermore, we aimed to compare the outcomes and complications of ETS for rrPAs and non-rrPAs.

## Methods

### Data Collection

After obtaining institutional review board approval and full patient consent, we collected data from consecutive patients diagnosed with rrPAs who underwent planned ETS in the Department of Neurosurgery of Xiangya Hospital, Central South University from January 2017 to June 2020. Besides, we included patients with non-rrPAs who underwent ETS in the same hospital from January 2019 to June 2020. All surgical procedures were performed by the senior authors ZY Li, and ZX Liu. Further, we retrospectively analyzed demographic, clinical, radiological, and surgical outcomes.

### Perioperative Evaluation

Pre- and post-operative hormone levels, including cortisol (8 am), adrenocorticotropic hormone (ACTH) (8 am), T3, T4, thyroid-stimulating hormone, prolactin, growth hormone, testosterone, estradiol, luteinizing hormone, and follicle-stimulating hormone, were examined in each patient. Prolactinoma, acromegaly, and Cushing disease were diagnosed as previously described (2, 24, 25). For patients with prolactinomas, remission was indicated by prolactin levels < 20 ng/mL and <15 ng/mL in women and men, respectively, on the day after surgery. For patients with growth hormone (GH)-secreting adenomas, biochemical remission was defined by a GH value < 0.4 ng/mL after a 75-g oral glucose load or a random GH value < 1.0 ng/ml and normal levels of insulin-like growth factor with adjustment for sex and age (19, 26). Remission of Cushing’s disease was indicated by early-morning cortisol levels of 1.8 mg/ml in the first 48 postoperative hours and normalization of the 24-h levels of urinary free cortisol (27). Ophthalmologists blinded to the surgery outcomes evaluated the vision and visual fields pre- and post-operatively. High-resolution and regular CT scans were used to evaluate the bony anatomy of the skull base before surgery and hematoma after surgery. All patients underwent contrast-enhanced magnetic resonance (MR) imaging preoperatively and 1–2 days postoperatively.

### Surgical Techniques

All surgical procedures were performed using an endoscopic transsphenoidal approach with a 0° and 30° rigid endoscope. For cases with a large portion of suprasellar extension or cavernous sinus invasion, an extended transsphenoidal or trans-cavernous sinus approach was applied as previously described (5, 28–32). Surgical navigation and mini-Doppler were routinely performed, especially in cases with tumor invasion in the cavernous sinus. A pedicled nasal mucosal flap was routinely formed, as appropriate. In case of an intraoperative high-flow CSF leakage, a fascia lata graft was harvested and placed in the sellar turcica, followed by covering with a pedicled mucosal flap, application of absorbable fibrin glue, and nasal packing. Postoperative low flow or high flow CSF leaks were evaluated not only based on the symptoms but also on the radiologic findings. In patients with no intraoperative CSF leak, if pneumocephalus presented on the CT after surgery, high-flow postoperative CSF leak was considered. While in patients with high-flow intraoperative CSF leak, If pneumocephalus on the CT scan increased or reoccurred several days after surgery, even a little amount of CSF discharge from the nose, high-flow postoperative CSF leak was also considered. In case of postoperative low-flow CSF leakage, conservative measures were applied, including 7-day bed rest with a head elevated 30 to 45 degrees and avoidance of nose blowing or coughing during lumbar drainage. Repair surgery was performed in case of postoperative high-flow CSF leakage that continued after lumbar drainage.

### Follow-up

Patients were followed up at 1, 3 and 6 postoperative months and subsequently annually. Neurological, endocrinological and MR images evaluations were performed for each patient. The extent of resection was evaluated on MRI at 3 postoperative months. A picture archiving and communication system was used to review all MR images. Two senior radiologists calculated the tumor volume using the ellipsoid model “(ABC)/2” equation. GTR, near-total resection, subtotal resection, and partial removal were indicated by removal of 100%, 90%–99%, 50%–90%, and < 50% of the tumor volume, respectively. For the functional pituitary adenomas, GTR was also required to meet the criteria of biochemical remission.

### Statistical Analysis and Propensity Score Matching

Continuous variables are presented as mean ± standard deviation and range. Categorical and continuous variables were analyzed using Pearson’s chi-square test and Mann-Whitney U-test, respectively. Logistic regression analysis was performed to determine independent predictive factors for postoperative CSF leakage. Statistical significance was set at p < 0.05. Statistical analyses were performed using SPSS 22.0 (IBM Corporation).

Postoperative outcomes were compared between patients with rrPAs and non- rrPAs using propensity score analysis. This methodology was applied to reduce between-group imbalance in the baseline patient characteristics. Based on covariates from the logistic model, we generated a propensity score for each patient in terms of age, sex, functional adenomas, tumor volume, and Knosp grade. Based on the inclusion criteria, 212 patients with non-rrPAs were consecutively recruited. We used a nearest-matching algorithm with a 1:1 ratio. Statistical analysis was performed using the R statistical program (version 4.4.0; R Core Team).

## Results

### Clinical Characteristics

According to the inclusion criteria, we enrolled 73 patients with rrPAs (mean age: 48.0±12.5 years). Among them, 41 (56.2%) and 14 (19.2%) presented with vision impairment and headaches. Moreover, 17 patients presented with endocrinological hyperfunction, including 3, 11, and 3 with prolactinomas, acromegaly, and Cushing’s disease, respectively. Additionally, 12 (16.4%) patients were asymptomatic upon admission (**Table 1**). With respect to initial surgery, 58 (79.5%) and 15 (20.5%) patients underwent initial endoscopic transsphenoidal surgery and microscopic transcranial surgery, respectively. The mean interval between initial and repeat surgery was 65.9 ±59.2 months. Six patients (8.8%) in the rrPA group received prior stereotactic radiotherapy and three patients (4.4%) had bromocriptine before repeat surgery. Upon reoperation, the mean tumor volume was 8.4 ±8.4 cm^3^. In our series, 57 (78.1%) and 16 (21.9%) patients had tumors of Knosp-Steiner grades 3–4 and 1–2 for invasion, respectively.

**Table 1:** Clinical characteristics of rrPAs.

Characteristics	No. of Patients (%)
Onset symptoms	
Visual loss**/**Visual field cuts	41 (56.2)
Headaches	14 (19.2)
Asymptomatic	12 (16.4)
Hormonal hyperfunction	17 (23.3)
Acromegaly	11 (15.1)
Prolactinomas	3 (4.1)
Cushing diseases	3 (4.1)
Preoperative hormonal deficiency	35 (47.9)
Single axis	23 (31.5)
Multiple axis	12 (16.4)
None	38 (52.1)
Extent of resection	
Gross total resection	41 (56.2)
Near-total resection	27 (37.0)
Subtotal resection	4 (5.5)
Partial resection	1 (1.4)

### Extent of Tumor Resection

**Figure 1:**
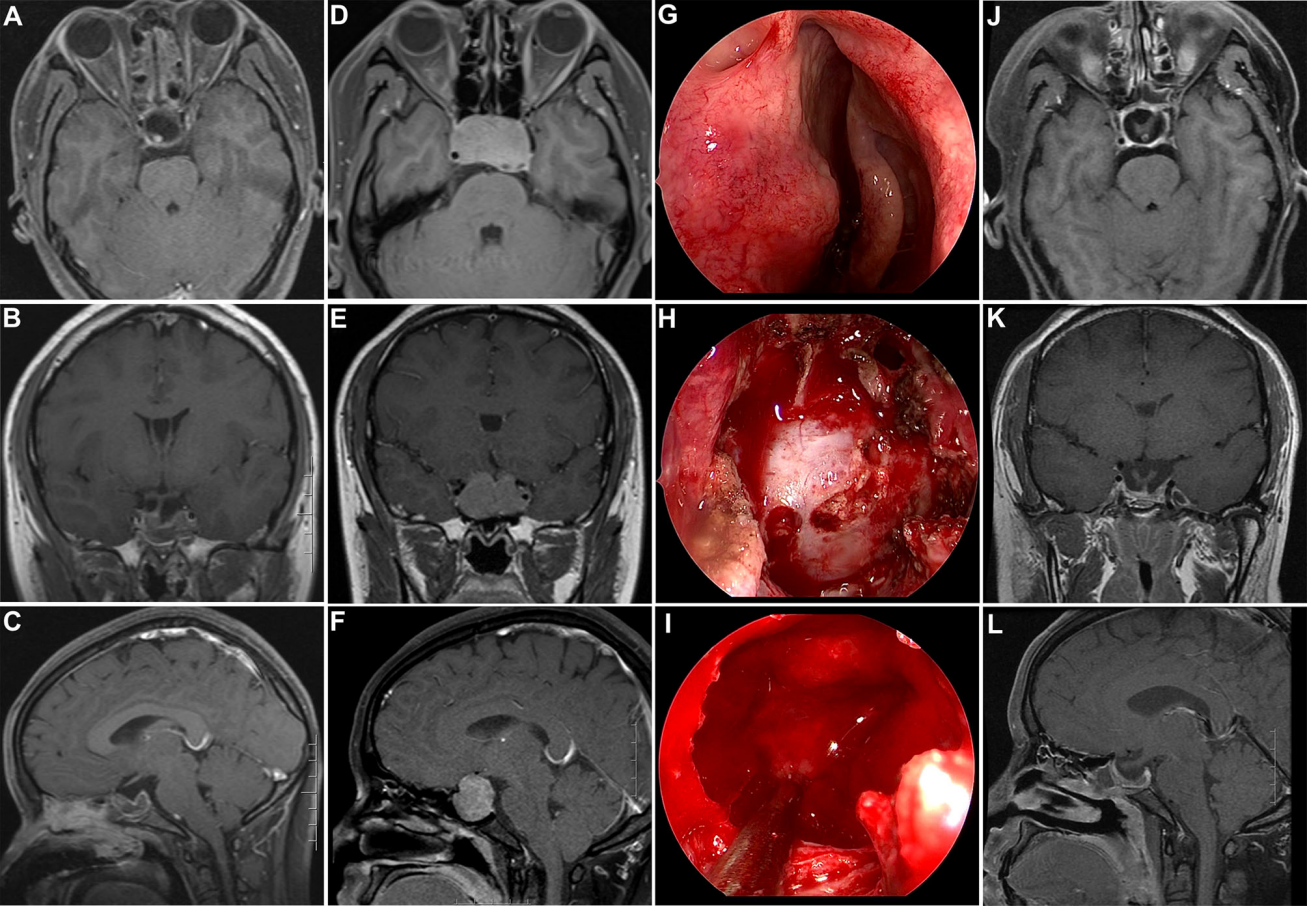
Illustrative case of a recurrent non-functional PA after prior transsphenoidal surgery. **(A–C)** Postoperative post-contrast T1-weighted MR images showing GTR after initial transsphenoidal surgery. **(D–F)** Post-contrast T1-weighted MR images showing tumor recurrence after 6 years. **(G–I)** Intraoperative photographs during repeat transsphenoidal surgery. **(H)** Wide sphenoidotomy was performed and a dural defect caused by the initial surgery was observed. **(I)** After tumor removal, the compressed pituitary and intact diaphragm sellae were displayed. **(J–L)** Post-contrast T1-weighted MR images acquired 3 months after repeated surgery showed complete tumor removal; additionally, both the pituitary and stalk were discernable.

**Figure 2:**
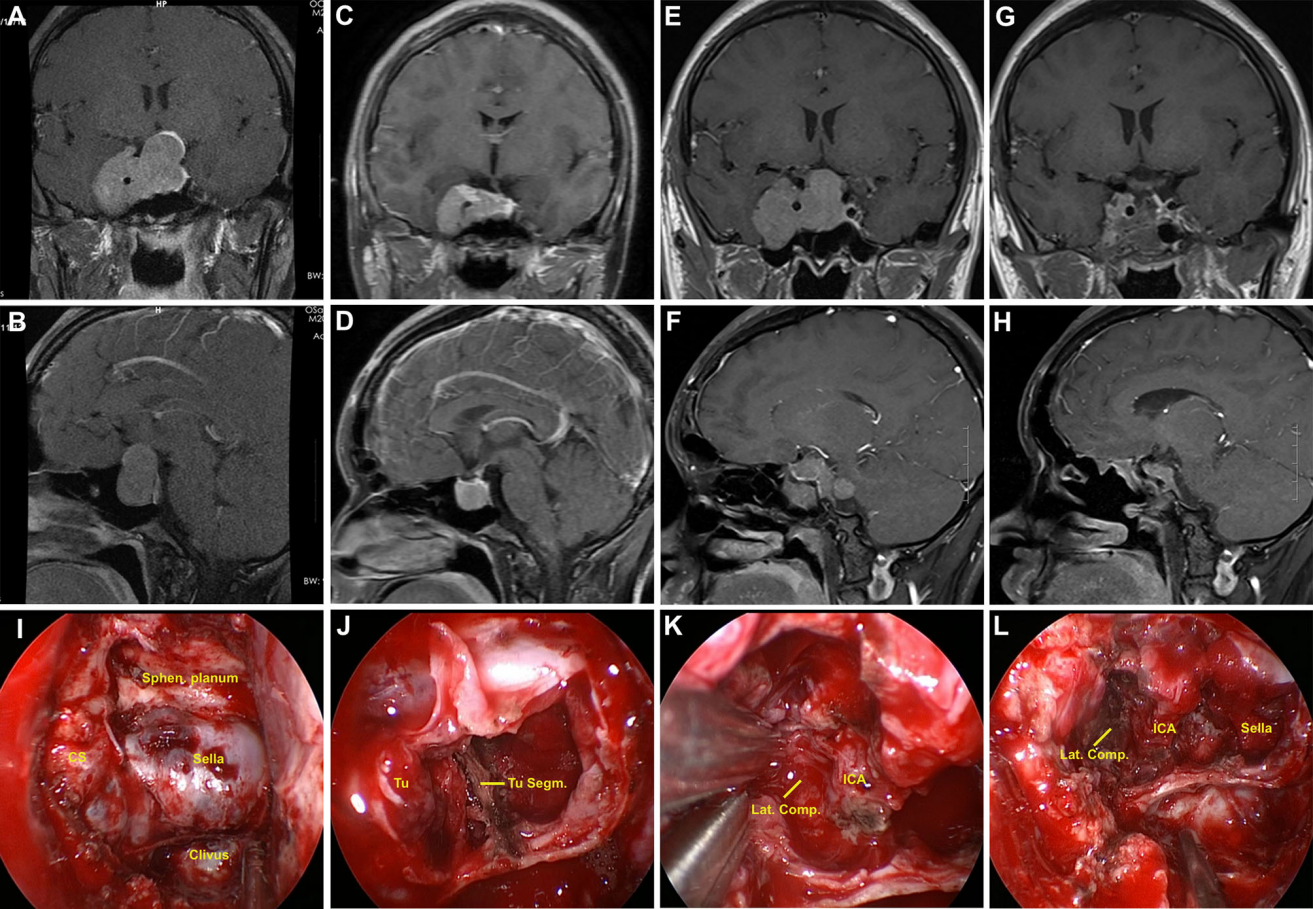
Illustrative case of a residual GH-secreting PA after prior transcranial surgery. **(A, B)** Preoperative coronal and sagittal post-contrast MR images showing a giant PA (Knosp grade 4), with the mass extending to the suprasellar area and cavernous sinus; moreover, cavernous segment of internal carotid artery (ICA) was totally encased by the tumor. **(C, D)** The patient had undergone transcranial surgery at another hospital. Early postoperative follow-up MR images revealed partial tumor removal. **(E, F)** Post-contrast T1-weighted MR images acquired one year after initial surgery showed residual tumor regrowth. **(G–H)** Postoperative MR images revealed near-total tumor removal. A small tumor piece was present at superior lateral compartment (Lat. Comp.)of cavernous sinus owing to adhesion. **(I–L)** Intraoperative photographs during repeat transsphenoidal surgery. **(I)** Sella and right cavernous sinus were widely exposed. **(J)** After debulking, tumor segmentation (Tu. Segm.) was observed. **(K)** Tumor in the right cavernous sinus presented fibrous and rubbery texture, and was sharply dissected from ICA. **(L)** Cavernous segment of ICA was observed after tumor removal. Sphen, sphenoidal.

Among the 73 rrPA patients, 41 (56.2%) achieved GTR (**Figure 1**), 27 (37.0%) underwent near-total resection (**Figure 2**), 4 (5.5%) underwent subtotal resection, and 1 (1.4%) underwent partial resection (**Table 1**). **Table 2** presents a comparison of the patients’ baseline characteristics between the GTR and non-GTR groups. Overall, there was no significant between-group difference in age and sex distributions. Among patients who achieved GTR in repeat surgery, 35 and 6 patients underwent prior transsphenoidal and transcranial surgeries, respectively. In the non-GTR group, 23 and 9 patients underwent prior transsphenoidal and transcranial surgeries, respectively. GTR showed a non-significant tendency to be achieved in patients with prior transsphenoidal surgery. The mean interval between the initial surgery and reoperation in the GTR and non-GTR groups were 59.3 ±38.1 and 74.3 ±78.4 months, respectively. Among functional PAs, GTR and non-GTR were achieved in nine and eight patients, respectively. The GTR group had a significantly lower mean tumor volume than the non-GTR group (6.2 ±7.2 cm^3^ vs. 11.1 ±9.1 cm^3^, p = 0.012). Proportion of high Knosp grade cases was found to be significantly higher in non-GTR group than in GTR group (93.8% vs 65.9%, p=0.002).

**Table 2:** Preoperative characteristics of rrPAs classified by GTR.

	No. of Patients (%)			
Characteristics	Total	GTR	Non-GTR	p Value
	73	41 (56.2)	32 (43.8)	
Gender				0.501
Male	31 (42.5)	16 (39.5)	15 (48.0)	
Female	42 (57.5)	25 (60.5)	17 (52.0)	
Mean age at op (yrs)	48.0 ± 12.5	47.9 ± 11.4	48.2 ± 14.0	0.903
Prior Approach				0.157
TSS	58 (79.5)	35 (85.4)	23 (71.9)	
TC	15 (20.5)	6 (14.6)	9 (28.1)	
Interval btwn initial & reop (mos)	65.9 ± 59.2	59.3 ± 38.1	74.3 ± 78.4	0.325
Function				
Functional	17 (23.3)	9 (22.0)	8 (25.0)	0.760
Non-functional	56 (76.7)	32 (78.0)	24 (75.0)	
Tumor volume (cm^3^)	8.4 ± 8.4	6.2 ± 7.2	11.1 ± 9.1	0.012
Knosp grade				0.002
0	5 (6.8)	5 (12.2)	0 (0.0)	
1	5 (6.8)	5 (12.2)	0 (0.0)	
2	6 (8.2)	4 (9.8)	2 (6.3)	
3 (3a,3b)	32 (43.8)	20 (48.8)	12 (37.5)	
4	25 (34.2)	7 (17.1)	18 (56.3)	

Op, operation; TSS, transsphenoidal surgery; TC, transcranial surgery; reop, reoperation.

### Preoperative Predictors for GTR of rrPAs

**Figure 3:**
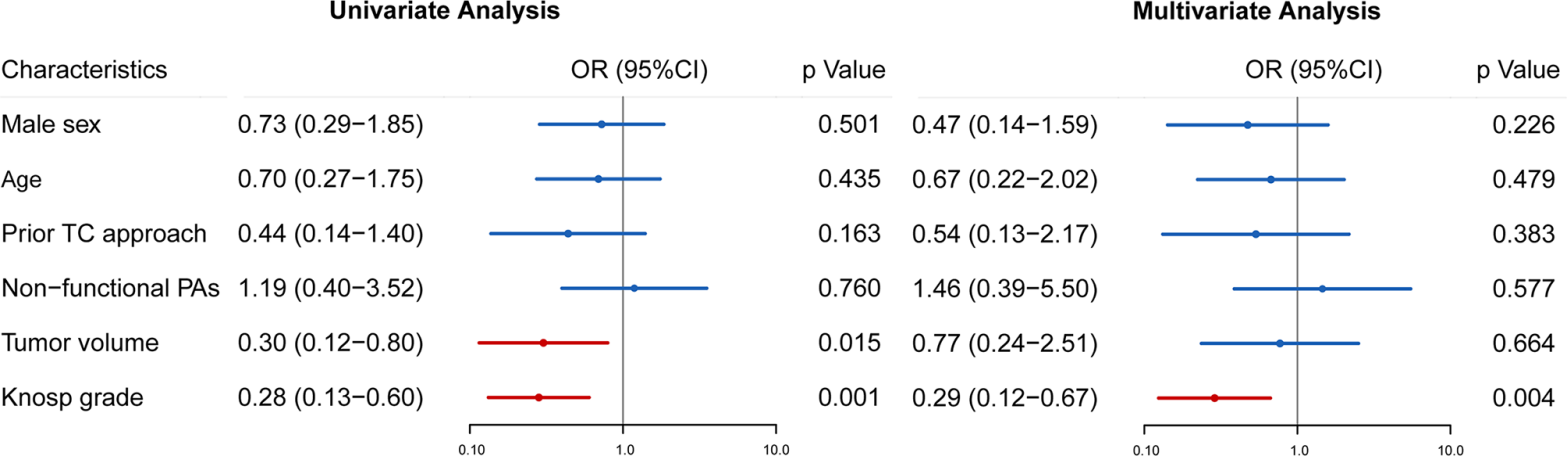
Univariate and multivariate analyses were performed to identify preoperative factors influencing GTR in patients with rrPAs. Odds ratio (OR), 95% confidence intervals (CI) and p value are exhibited for comparison.

To investigate factors related to the GTR rate in rrPAs, we performed logistic regression analysis with clinical characteristics, tumor volume, and Knosp grade. Univariate regression analysis revealed that patients with a larger tumor volume or higher Knosp grade had a higher risk of undergoing non-GTR. After adjustment for confounding factors in multivariate analysis, only Knosp grade was a significant adverse predictor (OR 0.324, 95% CI 0.147–0.715, p = 0.005), which indicated that tumors with higher Knosp grade are more difficult to achieve GTR (**Figure 3**).

### Clinical Outcomes

Among the 41 patients with preoperative vision impairment, vision status improved and remained stable in 9 (22.0%) and 30 (73.2%) patients, respectively. Two patients (4.9%) complained of postoperative vision deterioration, with both showing partial improvement after hyperbaric oxygen therapy during follow-up. At the final follow-up, 18 patients recovered from preoperative hypopituitarism and 5 patients developed hormonal deficiency requiring hormonal replacement therapy. Endocrinological remission was achieved in 8 cases, including 1, 5, and 2 with prolactinoma, GH-secreting adenomas, and ACTH-secreting adenomas, respectively. The other patient with ACTH-secreting adenoma showed temporary biochemical relief without residue on postoperative MRI. However, the patient developed Cushing syndrome at one postoperative year, with no mass being observed on dynamic contrast-enhanced MRI.

### Postoperative Complications

Postoperative CSF leakage occurred in five (9.6%) cases and ceased in five patients after lumbar drainage, with the remaining two patients undergoing repair surgery. Meningitis occurred in four cases and was successfully treated using antibiotics. Epistaxis occurred in four patients. Two patients were cured by nasal packing; the other 2 patients relieved after surgical treatment. Diabetes insipidus temporarily existed in 29 (39.7%) patients, with all patients showing relief during follow-up.

### Risk Factors for Intraoperative CSF Leakage of rrPAs

**Figure 4:**
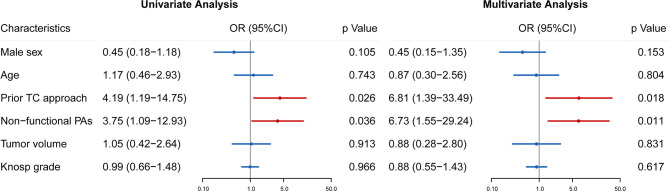
Univariate and multivariate analyses were used to identify risk factors for intraoperative CSF leakage in reoperation of rrPAs. Odds ratio (OR), 95% confidence intervals (CI) and p value are exhibited for comparison.

Almost all patients with postoperative CSF leakage experienced intraoperative CSF leakage. During reoperation, intraoperative CSF leakage occurred in 35 patients. We divided all the 73 patients into those with or without intraoperative CSF leakage. Patients with intraoperative CSF leakage had a longer postoperative hospital stay (12.5 ±10.9 days) than those without (8.1 ±5.8 days, p = 0.041). Logistic regression analyses were performed to determine the risk factors for intraoperative CSF leakage. Prior transcranial approach (OR 6.450, 95% CI 1.35511–30.696, p = 0.019) and non-functional adenomas (OR 7.472, 95% CI 1.562–35.737, p = 0.012) were significantly associated with intraoperative CSF leakage during reoperation (**Figure 4**).

### Outcomes and Complications Comparison Between rrPAs and Non-rrPAs

We then matched 73 cases of non-rrPAs from 212 patients with each case of the rrPAs group using propensity-score matching. After matching, there was no significant between-group difference in preoperative clinical characteristics. The rrPA group had a non-significantly higher GTR rate than the non-rrPA group (61.6% vs. 58.9%). There were no significant between-group differences in postoperative complications, including postoperative CSF leakage and meningitis. However, the rrPA group had a significantly higher incidence of intraoperative CSF leakage and a longer postoperative hospital stay than the non-rrPA group (p < 0.0001 and p = 0.017, respectively). Epistaxis was observed in four and no patients in the rrPA and non-rrPA groups, respectively. Among the patients who presented preoperative vision impairment, 24 (57.1%) and 9 (22%) patients in the non-rrPA and rrPA groups, respectively, showed postoperative vision improvement (p = 0.002). There was no significant between-group difference in the rate of hypopituitarism recovery or biochemical remission (**Table 3**).

**Table 3:** Preoperative Characteristics of non-rrPAs and rrPAs after propensity score matching.

	No. of Patients(%)			
Characteristics	Total	Non-rrPAs	rrPAs	p value
**Gender**	146	73	73	0.320
** Male**	68 (46.6)	37 (50.7)	31 (42.5)	
** Female**	78 (53.4)	36 (49.3)	42 (57.5)	
**Mean age at op (yrs)**	47.2 ± 13.7	46.5 ± 14.7	48.0 ± 12.5	0.499
**Function**				0.701
** Functional PAs**	36 (24.7)	19 (26.0)	17 (23.3)	
** Non-functional PAs**	110 (75.3)	54 (74.0)	56 (76.7)	
**Tumor volume (cm^3^)**	9.1 ± 9.9	9.8 ± 11.2	8.4 ± 8.4	0.383
**Knosp grade**				0.825
** 0**	11 (7.5)	6 (8.2)	5 (6.8)	
** 1**	8 (5.5)	3 (4.1)	5 (6.8)	
** 2**	10 (6.8)	4 (5.5)	6 (8.2)	
** 3 (3a,3b)**	62 (42.5)	30 (41.1)	32 (43.8)	
** 4**	55 (37.7)	30 (41.1)	25 (34.2)	
**Extent of resection**				0.501
** GTR**	86 (58.9)	45 (61.6)	41 (56.2)	
** Non-GTR**	60 (41.1)	28 (38.4)	32 (43.8)	
**Intraoperative CSF leak**	46 (31.5)	11 (15.1)	35 (47.9)	<0.0001
**Postoperative CSF leak**	9 (61.6)	2 (2.7)	7 (9.6)	0.275
**Meningitis**	6 (41.1)	2 (2.7)	4 (5.5)	0.681
**Epistaxis**	4 (2.7)	0 (0)	4 (5.5)	0.120
**LOS (days)**	8.8 ± 7.2	7.34 ± 4.9	10.2 ± 8.8	0.017
**Vision improved**	33/83 (39.8)	24/42 (57.1)	9/41 (22.0)	0.002
**Hypopituitarism recovered**	33/59 (55.9)	15/24 (62.5)	18/35 (51.4)	0.436
**Biochemical remission**	21/36 (58.3)	13/19 (68.4)	8/17 (47.1)	0.311
**Newly hypopituitarism**	9/87(10.3)	4/49 (2.0)	5/38 (13.2)	0.496

Op, operation; LOS, length of postoperative hospital stay.

## Discussion

### Surgical Outcomes

In our series, most symptomatic patients showed improvement after endoscopic transsphenoidal surgery. Specifically, 97.3% (71/73) of the patients showed improved/stable visual acuity while 51.4% of the patients recovered from preoperative hypopituitarism. Endocrinological remission was achieved in 47.1% of the patients. Do et al. reported biochemical remission in 9 out of 12 (75%) functional PAs and improved/stable visual acuity in 38 out of 40 (95%) non-functional PAs (33). Hwang et al. reported vision improvement in 19 (79%) patients (34). Our findings are generally comparable with these previous reports.

The average postoperative follow-up duration for patients with rrPAs was 29 months (range, 8–55 months). GTR was achieved in 42 (56.1%) patients, which is higher than previously reported values (14, 22, 33, 35, 36). Most tumors in re-operative patients were residual from the initial surgery rather than recurrence. For maximal resection of rrPAs, it is important to evaluate the location and reason of the residual tumor. In our series, residual tumors were more common in the parasellar and suprasellar regions after prior transsphenoidal surgery. Contrastingly, residual tumors were more common in the sphenoidal sinus and parasellar region after initial transcranial surgery. Inadequate opening of the sphenoid and sella is frequently observed. Therefore, for re-operation, we prefer wide sphenoidotomy and wide exposure of the sellar floor. For tumors extensively invading the supra-diaphragmatic region, the tuberculum sellae and planum were also removed. The anterior wall of the cavernous sinus was opened when the mass invaded the lateral compartment of the cavernous sinus (30). Moreover, the scar and tumor segmentation can impede the achievement of GTR during repeat surgery. Specifically, several tumor parts can be hidden by a tough fibrous septum to misguide the surgeon. It is important to thoroughly examine the tumor segmentation on preoperative MRI and to distinguish it from the normal boundary during surgery for complete mass removal. Doppler devices and precise neuronavigation facilitate accurate mapping of the carotid artery, as well as distinguishing the tumor compartment and anatomical boundary.

Preoperative factors influencing GTR remain unclear. The extent of resection is reported to be correlated with cavernous sinus invasion, tumor size, and initial surgery (endoscopic or microscopic transsphenoidal approach) (21, 33). Another study reported that Knosp-Steiner Grade 3–4 status was significantly associated with a non-GTR; however, GTR did not differ according to the prior approach (19). In our study, univariate analysis revealed a higher tumor volume and Knosp grade indicated a lower GTR rate. However, in the logistic regression model, only Knosp grade was an independent predictor of GTR. Tumors invading the lateral compartment of the cavernous sinus (Knosp grade 4) are challenging even with a trans-cavernous sinus approach, which could be attributed to the invasive tumor nature indicated by extension into the lateral compartment of the cavernous sinus and the resulting anatomical complexity (37, 38). Do et al. reported a GTR rate of 51.7% in patients who underwent reoperation for PA (70.3% and 21.7% for patients with Knosp grade 0–2 and 3–4, respectively) (33). In our cases, the GTR rate was 87.5% and 47.4% for patients with Knosp grade 0–2 and 3–4, respectively, which was higher than those in previous studies.

### Complications

Although there was a frequent occurrence of postoperative temporary DI in our series, all the patients were relieved during follow-up. Other complications of ETS include CSF leakage, meningitis, and epistaxis. Without prompt care, postoperative CSF leakage can cause severe outcomes, including meningitis or pneumocephalus. Intraoperative CSF leakage is reported to be an independent risk factor for postoperative CSF leakage (39–41). We found that prior transcranial surgery was an independent risk factor for intraoperative CSF leakage in re-operative ETS. Among patients who were previously treated with transcranial and transsphenoidal surgery, intraoperative CSF leakage occurred in 12 (80%) and 23 (39.7%) patients, respectively. This could be attributed to destruction of the diaphragm sellae and opening of the optic chiasmatic cistern during the prior transcranial surgery. Further, initial transcranial approach is generally chosen when the tumor has a large suprasellar component, where the residual tumor may remain. This may increase the risk of intraoperative CSF leakage during repeat surgery.

Given the morbidity of repeat transsphenoidal surgery, Radiation therapy including fractionated RT or stereotactic radiosurgery and medication (such as temozolomide) are also relatively safe treatment options for recurrent PAs.

### RrPAs Versus Non-rrPAs

Numerous reports have indicated that patients with rrPAs have a lower GTR and present with more complications than patients with non-rrPAs. However, most studies comparing GTR and complications between non-rrPAs and rrPAs were based on meta-analyses or previously published data (9, 11, 19, 32, 42). In this study, we used propensity score matching to mitigate between-group imbalance and found that the rrPA group had a significantly higher rate of intraoperative CSF leakage and longer postoperative hospital stay than the non-rrPA group. However, there was no between-group difference in the GTR rate and postoperative complications, which were inconsistent with previous findings. This could be attributed to the between-group differences in preoperative characteristic which may have resulted in outcome bias. Furthermore, the re-operations for rrPAs and surgery for non-rrPAs in previous studies were not performed by the same surgeons. Additionally, there are among-surgeon differences in techniques and experience, as well as among-institution differences in available armamentarium and materials. Yamada et al. also reported no difference in the complication rate between initial and repeat surgeries for rrPAs performed by experienced surgeons (11). Our study indicated that the extent of resection was affected by preoperative clinical features, including Knosp grade, but not by scarring and distorted anatomy caused by initial surgery. Although the rate of intraoperative CSF leak was higher for repeat surgery than for first-time surgery, a thorough multi-layer reconstruction technique could mitigate the rate of postoperative CSF leak after repeat surgery.

### Limitations

This study had several limitations. First, this was a single-institution study, potential bias may exist in patient selection for the different surgical procedures Second, although we included numerous clinical factors, several other preoperative characteristics including tumor segmentation, tumor lobulation, tumor fibrosis and Hardy stage were not analyzed in this study.

## Conclusion

The current study reported a series of re-operative endoscopic transsphenoidal surgeries performed on patients with rrPAs, which exhibited excellent outcomes and relatively low complication rates. Knosp grade was an independent predictor of GTR in repeat transsphenoidal surgeries. Previous transcranial surgery and non-functional PAs were significantly associated with intraoperative CSF leakage during reoperation. There is a need for careful decision-making for patients with previous transcranial surgery or non-functional PA regarding undergoing revision transsphenoidal surgery in case of CSF leakage. Compared with initial surgery, endonasal endoscopic reoperation allows comparable outcomes and complication rates.

## Data Availability Statement

The raw data supporting the conclusions of this article will be made available by the authors, without undue reservation.

## Ethics Statement

Studies involving human participants were reviewed and approved by Medical Ethics Committee of the Xiangya Hospital, Central South University (No.201612816). Written informed consent was obtained from the individual(s) for publication of any potentially identifiable images included in this article.

## Author Contributions

Conception and design: XG and ZXL. Acquisition of data: HSZ, YZ, and HCY. Analysis and interpretation of data: HSZ and XG. Drafting the article: HSZ and XG. Critically revising the article: XG, HSZ and ZXL. Statistical analysis: XG and HSZ. Administrative/technical/material support: CTL, KY, SSF, MYZ, and ZYL. Study supervision: ZXL. All authors contributed to the article and approved the submitted version.

## Funding

This study was supported by the National Natural Science Foundation of China (grant No. 81701285, 81873635, 82172685) and the Natural Science Foundation of Hunan Province (grant No. 2018JJ3824).

## Conflict of Interest

The authors declare that the research was conducted in the absence of any commercial or financial relationships that could be construed as a potential conflict of interest.

## Publisher’s Note

All claims expressed in this article are solely those of the authors and do not necessarily represent those of their affiliated organizations, or those of the publisher, the editors and the reviewers. Any product that may be evaluated in this article, or claim that may be made by its manufacturer, is not guaranteed or endorsed by the publisher.
